# Daytime Radiative Cooling Sheet Functionalized by Al_2_O_3_‐Assisted Organic Composite

**DOI:** 10.1002/advs.202417584

**Published:** 2025-02-03

**Authors:** Jaein Park, Dongwoo Chae, Hangyu Lim, Jisung Ha, Seongwoo Park, Hansang Sung, Chanwoong Park, Heon Lee

**Affiliations:** ^1^ Department of Materials Science and Engineering Korea University Anam‐ro 145 Seongbuk‐gu Seoul 02841 Republic of Korea; ^2^ ZERC, 620 New Engineering Building 73‐15, Anam‐ro Seongbuk‐gu Seoul 02855 Republic of Korea

**Keywords:** atmospheric transparency window, daytime radiative cooling, electrospinning, microfibers, solar reflectance

## Abstract

Daytime radiative cooling presents a compelling technology, noted for its efficiency and environmental friendliness. Recent studies have focused on not only the cooling capacity but also the applicability and versatility of this technology. This study introduces a daytime radiative cooler as a sheet with exceptional cooling performance. Its matrix is composed of polymethylmethacrylate (PMMA) and thermoplastic polyurethane (TPU), which are emerging organic materials suitable for radiative cooling. Furthermore, aluminum oxide (Al_2_O_3_) is employed as a supporting dielectric particle to enhance cooling performance. An Al_2_O_3_‐assisted organic composite (AOC) is created through electrospinning and hot‐pressing, resulting in a bendable sheet form. The AOC sheet demonstrates a light reflectance of 97.9% across the solar spectral region (0.3–2.5 µm) and an emissivity of 95.2% within the atmospheric transparency window (ATW) of 8–13 µm. The cooling power, derived from optical properties, is calculated to be 120.1 Wm^−2^. Experimental findings confirm the AOC sheet's capability to achieve 4.9 °C below ambient temperature and, when applied to a car model, to reduce the interior temperature by 12.7 °C.

## Graphical abstract

Radiative coolers composed of polymer matrix incorporating dielectric particles are actively developed due to their excellent optical properties and mechanical characteristics, offering versatility in applications. Based on the internal properties of the comprising materials, a daytime radiative cooler in the form of an aluminum oxide (Al_2_O_3_)‐assisted organic composite sheet with exceptional cooling performance along with applicability is introduced.

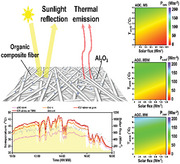

## 1 Introduction

Space cooling and heat dissipation are critical issues amid the current state of global warming.^[^ ^1^, ^2^ ^]^ Since the onset of the Third Industrial Revolution, the Earth has been subjected to consistent heating, with recent concerns focusing on greenhouse gas emissions and the emergence of urban heat islands as global challenges.^[^ ^3^, ^4^, ^5^, ^6^ ^]^ Various strategies have been proposed to manage heat sources impacting climate change and prevent further warming of the Earth. However, most existing cooling technologies are energy‐intensive, particularly in their electricity use. Air conditioning, a prevalent cooling approach, contributes to a feedback loop exacerbating cooling and warming cycles due to the significant emissions of greenhouse gases, like carbon dioxide, associated with electricity generation. Consequently, there is a pressing need to develop and adopt new cooling methodologies.

Radiative cooling has emerged as a promising technology, garnering increasing interest because of its distinct advantages over traditional cooling methods.^[^ ^7^, ^8^, ^9^, ^10^ ^]^ Its most notable feature is its eco‐friendliness, operating without energy consumption, thus eliminating concerns related to the feedback loop. Daytime radiative cooling is advantageous during peak air temperatures. This energy‐free operation is facilitated by leveraging the intrinsic optical properties of materials comprising the radiative cooler. It is designed to exhibit high light reflectance within solar wavelengths (0.3–2.5 µm), thus effectively blocking incoming solar heat.^[^ ^11^, ^12^ ^]^ Additionally, minimizing solar absorption is crucial to avoid solar heating. A radiative cooler must also efficiently emit heat within the atmospheric transparency window (ATW) corresponding to (8–13 µm),^[^ ^13^, ^14^ ^]^ allowing for the dissipation of absorbed heat into cold outer space as infrared (IR) energy. Thus, spectral selectiveness, characterized by high solar reflectance and IR emissivity, is vital for superior cooling performance.

Previous research has identified materials that meet the optical specifications for effective radiative cooling, including metamaterials,^[^ ^15^, ^16^ ^]^ photonic crystals,^[^ ^17^, ^18^, ^19^, ^20^ ^]^ metals,^[^ ^21^, ^22^ ^]^ and polymers.^[^ ^23^ ^]^ These materials have facilitated the development of radiative coolers in forms such as photonic films,^[^ ^24^ ^]^ multilayer structures,^[^ ^18^ ^]^ and alternating layers, utilizing the requisite optical properties to achieve efficient cooling. However, the application of these materials is limited. As a result, there has been a growing demand for radiative coolers that balance cooling efficiency with versatility. Recent efforts have led to the development of radiative coolers with simple structures, such as single‐layer sheets,^[^ ^25^, ^26^ ^]^ paint‐like formats,^[^ ^14^, ^27^, ^28^, ^29^ ^]^ and polymeric films,^[^ ^30^, ^31^, ^32^, ^33^, ^34^ ^]^ which are easier to manufacture, cost‐effective, and broadly applicable.

In the structure of a radiative cooler, particularly in the case of a sheet,^[^ ^35^, ^36^, ^37^ ^]^ there is an advantage that it yields not only high cooling performance but also applicability. However, previous studies on radiative cooling sheets often use expensive polymer materials including polyvinylidene fluoride (PVDF), polydimethylsiloxane (PDMS), polyimide (PI), and poly(vinylidene fluoride‐co‐hexafluoropropylene) (P(VDF‐HFP)), and costly, difficult‐to‐handle nanoparticles. Further, the fabrication process is often complex and involves multiple steps. Moreover, although previously developed radiative cooling sheets possess excellent cooling capabilities, most of them still need more optimization in optical properties and hold a considerable degree of solar absorption. Thus, there is still potential for further improvement.

This study unveils a novel daytime radiative cooling sheet using relatively inexpensive polymer materials and dielectric particles through a simple process of stirring, electrospinning, and hot‐pressing. This innovative sheet comprises a network of organic composite microfibers interwoven with embedded dielectric ceramic particles. The organic composite is primarily formed from polymethylmethacrylate (PMMA) and thermoplastic polyurethane (TPU), with aluminum oxide (Al_2_O_3_) nanoparticles randomly distributed throughout the fiber matrix. For enhanced cooling performance, the synergistic combination of these materials was optimized to maximize solar reflectance and mid‐infrared emissivity, which are crucial for effective daytime radiative cooling. This design allows for scalable and cost‐effective fabrication, making it a promising solution for passive cooling applications in extreme climate conditions.

## 2 Results and Discussion

Building on the unique properties of PMMA, TPU, and Al_2_O_3_, an Al_2_O_3_‐assisted organic composite (AOC) sheet has been developed to achieve optimal radiative cooling performance. PMMA, known for its widespread application in various sectors, including radiative cooling, is chosen for its inherent properties and cost‐efficiency. It exhibits a negligible extinction coefficient (k) across the solar spectrum, effectively reducing solar absorption.^[^ ^38^, ^39^ ^]^ Additionally, PMMA features multiple vibrational modes,^[^ ^40^ ^]^ which enhance its IR absorptivity/emissivity within the ATW. However, PMMA alone has limitations such as brittleness, reduced toughness, and lower impact resistance, which may restrict its mechanical durability in practical applications.

To overcome these drawbacks, TPU, recognized for its durability, high strength, and cost‐effectiveness, is introduced into the composite matrix. TPU has been widely adopted in various applications, including radiative cooling,^[^ ^41^, ^42^ ^]^ due to its mechanical robustness and adaptability to different substrates. The combination of PMMA and TPU in this work results in an organic composite matrix that not only exhibits superior optical properties but also offers enhanced versatility and applicability.

To examine the spectral properties of PMMA and TPU, ATR‐FTIR analysis was conducted, with the fully fabricated AOC sheet also included to confirm the effective incorporation of both matrix materials, as shown in Figure S1 (Supporting Information). Both PMMA and TPU exhibit multiple absorption peaks in the ATW region, highlighting their ability to efficiently absorb and emit thermal radiation within the IR range. The absorption peaks in the ATW region, associated with the vibrations of several functional groups including C─O, and O─CH_3_ bonds, contribute to the matrix's strong emissivity within the ATW, making it a critical component in enhancing the radiative cooling performance. The functional groups further improve the matrix's radiative properties, ensuring efficient heat dissipation. The combined spectral contributions of PMMA and TPU ensure significant absorption and emission in this critical range, allowing the designed AOC sheet to function effectively even under intense solar irradiation.

Further, Al_2_O_3_, specifically chosen as a ceramic dielectric particle, is incorporated to augment the cooling efficiency by increasing solar light scattering and minimizing solar heating. Due to its highly refractive property and broadband scattering capabilities, Al₂O₃ enhances the sheet's ability to reflect incident solar radiation, thereby reducing overall heat absorption. Al₂O₃ particles also exhibit absorption peaks that partially overlap with the ATW, contributing to the composite's thermal radiative properties. However, these peaks were not explicitly shown in the ATR‐FTIR analysis. The primary reason for this exception lies in the inherent limitations of ATR analysis when applied to particulate materials like Al₂O₃. Due to their physical nature, Al₂O₃ particles tend to produce inconsistent and less reliable intensity values, introducing significant measurement errors. To address this, the focus of the ATR‐FTIR analysis was placed on the PMMA and TPU polymer matrix, where the spectral data are more accurate and reproducible. The synergistic effect of all the components results in optimizing the optical properties of the AOC sheet for radiative cooling.

**Figure 1:**
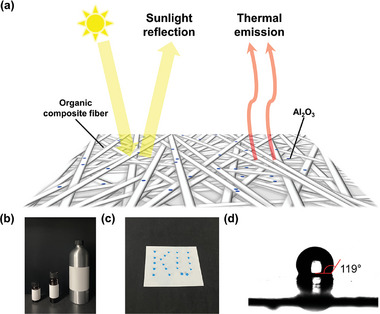
a) Schematic of the AOC sheet with its components and cooling mechanism, b) photo image of the AOC sheets covered on curved objects, c) photo image of the AOC sheet with dyed water droplets, aligned to express the initial “KU”, and d) water contact angle of the AOC sheet, measured to be 119°, confirming the hydrophobicity of the sheet. Water contact angle values represent the average of three separate measurements (*n* = 3), and data are expressed as mean ± standard deviation (SD). Statistical comparison was performed using an independent *t*‐test (*p* < 0.05).

A schematic detailing the sheet structure and radiative cooling mechanisms is shown in **Figure** **1**a. The polymeric nature of the composite matrix enables the application of the AOC sheet across a diverse array of objects and uses including curved surfaces, as depicted in Figure 1b. Beyond its optical properties and adaptability, the hydrophobic quality of the sheet (Figure 1c), attributed to its smooth surface finish, is a significant advantage for practical applications. This hydrophobicity is quantitatively demonstrated through the precise measurement of the water contact angle, as shown in Figure 1d.

**Figure 2:**
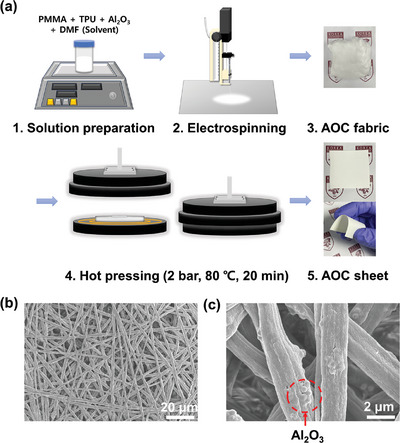
a) Entire fabrication process of the AOC sheet, including the electrospinning process to produce the AOC fabric and the subsequent hot‐pressing to alter the fabric into a sheet, SEM image of b) low and c) high resolution. Microstructural analysis was performed on five different regions per sample (*n* = 5), and representative images are shown.

The manufacturing process of the AOC sheet involves mixing PMMA, TPU, and Al₂O₃ in N,N‐Dimethylformamide (DMF) solvent to create a solution (**Figure** **2**a). This solution is then electrospun onto a stainless use steel (SUS) plate, producing a soft, fluffy AOC fabric. Despite successfully fabricating the AOC fabric with Al₂O₃‐embedded microfibers into the desired structure, its practical application is limited due to handling difficulties. The microfibers are not densely networked, making them prone to separation and stripping from the surface, and the fiber layers can peel off with minimal contact. Additionally, the optical properties of the AOC fabric fall short for radiative cooling purposes, attributed to insufficient solar reflectance and inadequate IR emission efficiency. These limitations result from the sparse fiber distribution and excessive vacant areas.

To overcome these drawbacks and optimize the structure for cooling, the fabric undergoes a hot‐pressing process. The combined application of heat and pressure consolidates the microfiber distribution and compacts the fiber network, resulting in a more stable structure. The microstructure of the AOC sheet, depicted in Figure 2b,c, shows that the Al_2_O_3_‐embedded organic fibers are more densely arranged without altering their shapes. This denser arrangement creates additional solar scattering sites, enhancing solar reflectance and the potential for heat radiation via IR emission. The microstructural comparison of the AOC fabric before and after hot‐pressing, along with the differences in optical properties, are illustrated in Figure S2 (Supporting Information). Further, the numerically averaged optical properties of the two comparatives are indicated in Table S1 (Supporting Information).

**Figure 3:**
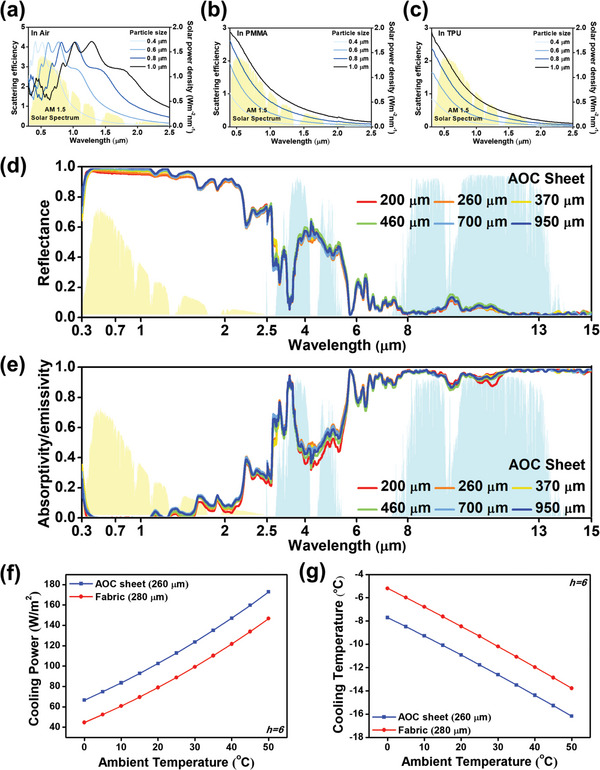
Scattering efficiency of Al_2_O_3_ particles of different sizes in a) air, b) PMMA, and c) TPU, d) measured solar reflectance, e) spectral emissivity, f) calculated cooling power, and g) cooling temperature of the AOC sheet and fabric regarding the ambient temperature, under the condition of h = 6 W m^−2^ K^−1^, 888 W m^−2^ of solar irradiance and the ideal AM1.5 spectrum. Spectral data were derived from three independent measurements (*n* = 3) per sample and are presented as mean ± SD. The statistical difference between the AOC sheet and fabric was determined via an independent *t*‐test (*p* < 0.05).

Al₂O₃, with a bandgap energy of ≈9 eV, which is significantly higher than that of solar photons (≈4.13 eV) exhibits minimal solar absorption.^[^ ^43^, ^44^ ^]^ Its high refractive index (n) and low extinction coefficient (k) across solar wavelengths, shown in Figure S3a (Supporting Information), contribute to elevated solar reflectance and reduced sunlight absorption.^[^ ^45^ ^]^ Conversely, in the IR spectrum, particularly within the 10–13 µm range, Al₂O₃ demonstrates a high k/n ratio, enhancing IR emissivity. The selected Al₂O₃ particles, ranging from nano to micro size (Figure S3b, Supporting Information), influence the optical behavior in the solar spectrum by diffracting more solar light within the composite. The lattice alignment and crystallinity of these particles are confirmed through XRD 2θ plots (Figure S3c, Supporting Information). The embedded Al_2_O_3_ particles further enhance the optical behavior of the composite by promoting solar scattering in both the vacant areas and among the organic fibers. The Mie‐scattering efficiencies for Al₂O₃ particles, with the most common size distribution in air, PMMA, and TPU, aligned with the solar spectrum of corresponding wavelengths, are illustrated in **Figure** **3**a–c. Analysis reveals that the scattering peaks of Al_2_O_3_ particles in these mediums align with regions of the highest solar power density, optimizing solar scattering efficiency. To determine the optimal Al₂O₃ content in the AOC sheet, the weight percentage (wt%) of Al₂O₃ was varied. Samples with 0, 5, 10, and 15 wt% Al₂O₃ were analyzed for optical characteristics and microstructure. The 10 wt% Al₂O₃ sample exhibited the most favorable composition, achieving optimized optical properties for radiative cooling. Consequently, the AOC sheet was designed to contain 10 wt% Al₂O₃. The microstructures of the AOC sheets with 0, 5, and 15 wt% Al₂O₃, along with their spectral characteristics, are displayed in Figures S4 and S5 (Supporting Information), respectively. Solar reflectance and IR emissivity for each sample are detailed in Table S2 (Supporting Information).

Theoretical and experimental analyses focused on the composite's constituents and structure, leading to the fabrication of AOC sheets of varying thicknesses to evaluate thickness impact on optical properties. The spectral reflectance and absorptivity/emissivity of the AOC sheets of different thicknesses are shown in Figure 3d,e. According to calculations, sheets of 200, 260, 370, 460, 700, and 950 µm exhibited 94.7%, 95.0%, 96.3%, 96.9%, 97.2%, and 97.9% of solar reflectance. Solar absorption of the sheets of all thicknesses was calculated to be extremely low, considering that even the sheet with the highest thickness, 950 µm, exhibited only 1.3% absorptivity. Reflectance increased with thickness, while solar absorption stayed minimal. The AOC sheet's optical properties in the solar spectrum suggest significant radiative cooling potential, even at thicknesses below 270 µm, with enhancements as thickness increases. This can be achieved by using more solutions in the electrospinning process. Absorptivity remained low in the solar spectrum for all sheets but increased significantly within the IR spectrum, including the ATW. In contrast, reflectance increased, while broadband transmittance shown in Figure S6 (Supporting Information) remained negligible, regardless of sheet thickness. The vibrational modes of PMMA and the high k value of Al₂O₃ in the IR spectrum contribute to the sheets' effectiveness in radiating heat into space.^[^ ^40^, ^44^ ^]^ The AOC sheets, from the lowest to the highest thicknesses, showed 94.7%, 95.8%, 95.5%, 94.4%, 95.4%, and 95.2% of calculated absorptivity/emissivity in the ATW, proving the adequate radiative properties for heat dissipation and cooling. The IR emissivity showed minor differences across thicknesses, attributed to the inherent properties of the materials. The cooling power of the thickest sheet, 950 µm, was calculated to be 120.1 Wm⁻^2^, under the AM1.5 global spectrum, with a heat transfer coefficient (h) of 6 Wm^−2^ K^−1^ and an ambient temperature of 300 K.

To validate the cooling efficacy of the AOC sheet, its cooling power and temperature reduction capabilities were compared to those of a commercial fabric of similar thickness under various ambient temperatures (illustrated in Figure 3f,g). It was observed that cooling power increases proportionally to ambient temperature, indicating a more pronounced cooling effect in hotter environments. Independent of ambient temperature, the AOC sheet consistently exhibited a cooling power exceeding that of the fabric by more than 22.0 Wm⁻^2^. Additionally, temperature simulations suggested that the AOC sheet could achieve a cooling effect greater than 2.4 °C than the fabric. These findings, based on measured properties and simulation results, affirm the superior cooling potential of the AOC sheet relative to existing covering materials.

**Figure 4:**
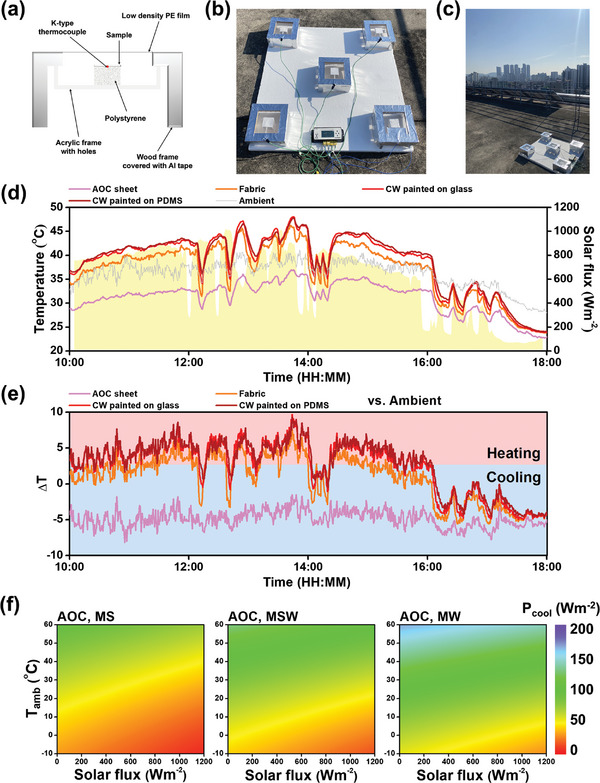
a) Schematic of the outdoor measuring chamber, photo image of b) close view and c) distant view of the outdoor experiment setup, d) temperature trends during the experiment, e) temperature difference between the samples and ambient. f) Contour color maps of the cooling power of the AOC sheet in MS, MSW, and MW, with respect to the solar flux and ambient temperature. Temperature measurements were conducted over four independent trials (*n* = 4), and data are presented as mean ± SD. Statistical significance among different samples was assessed using one‐way ANOVA followed by Tukey's post‐hoc test (*p* < 0.05).

To evaluate the actual cooling performance of the AOC sheet, an outdoor temperature measurement experiment was conducted in Seoul, South Korea.^[^ ^46^, ^47^, ^48^, ^49^, ^50^ ^]^ This experiment involved using an outdoor measuring chamber, the schematic of which is shown in **Figure** **4**a.^[^ ^51^ ^]^ The chamber's main structure was constructed from wood, with an acrylic frame attached to the wooden base. Holes were drilled into the acrylic frame's walls to mitigate internal heat accumulation. Additionally, polystyrene foam was placed on top of the acrylic frame to support the measured sample and reduce heat transfer between the interior of the frame and the sample. The exterior of the wooden structure was wrapped in aluminum tape to decrease solar absorption and prevent heat buildup on the chamber's surface. A thin layer of low‐density polyethylene (LDPE) film covered the chamber's top, positioned 1 cm above the polystyrene foam, as a barrier against conduction and convection from the external environment to the sample. A K‐type adhesive thermocouple was positioned between the sample and the polystyrene foam to ensure accurate temperature measurement with minimal error and was firmly secured.

For ambient temperature measurements, a K‐type copper wire thermocouple was situated beneath the acrylic frame of the chamber in an area without a convection shield. Additionally, all measuring chambers were placed on a 5 cm‐thick polystyrene board to avoid heat transfer from the ground. Temperature data were captured using a temperature data logger, while meteorological data were gathered from a weather station, as depicted in Figure S7 (Supporting Information). This comprehensive setup provided a controlled environment for accurately assessing the AOC sheet's cooling ability under real‐world conditions.

For the collective temperature data of the AOC sheet, the sheet of 950 µm was attached to the polystyrene foam as the sample to be measured. For comparative samples, a bare fabric was also measured along with a glass substrate and PDMS sheet coated with commercial white (CW) paint. The thickness of the glass and PDMS was 500 and 1100 µm, respectively. The spectral optical properties of the comparative samples are shown in Figure S8 (Supporting Information), and the average values are indicated in Table S3 (Supporting Information). The site of the entire measurement is in Figure 4b. Another view of it with the sky condition shown is in Figure 4c. The measured temperature is shown in Figure 4d, with solar flux present during the measurement. Results indicated that the average temperature of the AOC sheet, fabric, CW‐paint‐coated glass, and PDMS were measured to be 31.3, 37.1, 38.7, and 39.0 °C, each by each. The average value of the measured ambient temperature during the outdoor experiment was 36.2 °C and the average solar flux was 612.6 Wm^−2^. The recorded weather data is shown in Figure S9 (Supporting Information). All the monitored weather data significantly affect the radiative cooling performance. It was observed that the AOC sheet was cooled by an average of 4.9 °C and up to a maximum of 8.2 °C in relation to the outer ambient. Further, the average temperature of the AOC sheet was 5.8 °C lower than the fabric and lower by over 7 °C than the CW‐paint‐coated substrates, demonstrating significantly superior cooling capabilities to commonly used covers that are applied on objects. To observe the sub‐ambient cooling capacity of each sample, a temperature comparison of the samples versus the outdoor ambient is displayed in Figure 4e. Apart from the AOC sheet, all the samples exhibited higher temperatures than the ambient, resulting in heating when the solar flux was most intense.

Notably, between 11:00 and 12:00, the period with maximum solar flux averaging 831.7 Wm⁻^2^, the average temperature of the AOC sheet continued to cool at 4.7 °C below the ambient temperature, whereas the fabric, CW‐paint‐coated glass, and PDMS experienced temperature increases of 3.2, 5.1, and 5.3 °C, respectively, relative to the ambient temperature. The temperature measurement results corresponding to this period are shown in Figure S10 (Supporting Information). This consistent sub‐ambient cooling capability of the AOC sheet regardless of solar intensity emphasizes its exceptional efficiency and potential advantages for cooling applications, distinguishing it from conventional covering materials. Further, from these results, it is strongly expected that the cooling power and performance will be much more prominent when the atmospheric transmittance is higher.

Further, from these results, it is strongly expected that the cooling power and performance will be much more prominent when the atmospheric transmittance is higher.

When the ambient measuring thermocouple was positioned in the exact location as the samples, under conditions where the LDPE film inhibited conduction and convection, the AOC sheet exhibited a 9.5 °C cooler than the corresponding inner ambient temperature. This inner ambient temperature, gauged under identical conditions to the samples and with non‐radiative sources mitigated by weather conditions, was higher than the external ambient temperature. Consequently, the temperature differential in this scenario was more pronounced. The measured temperature profile and the comparison accounting for the inner ambient temperature are illustrated in Figure S11 (Supporting Information).

Given the myriad factors affecting radiative cooling, such as geographic location, weather, and seasonality, the assessment of cooling performance must be nuanced, considering the specific conditions under which a radiative cooler operates. Contour color maps showcasing the simulated cooling power of the AOC sheet across different seasons in mid‐latitude regions are presented in Figure 4f. These maps utilize MS, MSW, and MW abbreviations to denote mid‐latitude summer, the transitional seasons between summer and winter (spring and autumn), and winter, respectively. The maps are scaled by solar flux and ambient temperature, with the color gradient indicating cooling power, ranging from red for 0 Wm^−2^ to violet for 200 Wm^−2^. The progression from red to purple signifies an increase in cooling effect. Notably, within a given season, an increase in solar flux corresponds to a decrease in cooling power, as reflected by a downward shift in the color gradient on the map. Moreover, the seasonal transition from summer through spring and autumn to winter is depicted by a gradual shift in the color distribution from red to violet, indicating variances in cooling efficiency.

**Figure 5:**
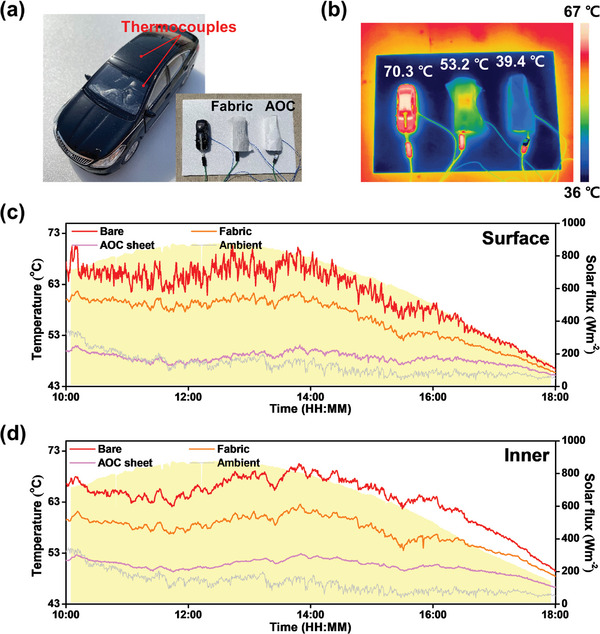
a) Photo images of the car model and the temperature measurement setup of the models under different conditions, b) IR image of the car models during the measurement, and temperature changes at the c) surface and d) inner. Three independent trials (*n* = 3) were conducted for outdoor temperature monitoring, with data presented as mean ± SD. Differences in surface and interior temperatures between the models were statistically evaluated using one‐way ANOVA with Tukey's post‐hoc test (*p* < 0.05).

To showcase the AOC sheet's real‐world applicability, an external temperature measurement experiment was conducted in Dubai, United Arab Emirates, using three identical car models under different conditions. One car model was left in its original state without any modifications, while the other two were covered with commercial fabric and the AOC sheet, respectively. To minimize heat conduction between the models and the ground, each was placed on a 1 cm‐thick polystyrene board. Temperature measurements were taken on both the upper surface and inside each vehicle model. An adhesive thermocouple was affixed to the exact location on the roof of each car to measure surface temperature, and a wire thermocouple was placed on the driver's seat to monitor the interior temperature changes. Outdoor ambient temperature was measured with a copper wire thermocouple housed inside a bottle covered with aluminum tape to permit convection and conduction, ensuring the thermocouple's tip did not contact the bottle's surface. This setup allowed for reliable air temperature measurements during the experiment. **Figure** **5**a displays a close view of the vehicle model indicating the measuring spots, along with an image of the entire setup.

The experiment used infrared (IR) imaging to compare temperatures across the models (Figure 5b). The uncovered car model registered a temperature of 70.3 °C, the fabric‐covered model showed 53.2 °C and the model with the AOC sheet exhibited 39.4 °C. Real‐time temperature changes for both the surface and interior of the car models are presented in Figure 5c,d. Under an average solar flux of 651.1 Wm⁻^2^, the surface temperature of the bare car averaged 63.6 °C. The cars covered with fabric and the AOC sheet recorded surface temperatures of 57.4 and 50.5 °C, respectively, while the interior temperatures were 61.2 °C for the bare model, 56.3 °C for the fabric‐covered, and 48.5 °C for the AOC sheet‐covered vehicle. The measured average outdoor ambient temperature was 47.1 °C.

Although the cooling performance, as depicted in the IR image, was less pronounced, the results confirmed significant cooling benefits when using the AOC sheet. The car's surface covered by the AOC sheet cooled by 13.1 °C in relation to the surface of the bare car model. More notably, the interior of the AOC sheet‐covered car was 12.7 °C cooler than that of the bare model. Compared to the commercial fabric, the AOC sheet achieved an additional surface cooling of 6.9 °C and an interior cooling of 7.8 °C, underscoring the AOC sheet's superior cooling capabilities.

During the peak solar flux period from 10:00 to 14:00, the cooling performance of the AOC sheet was notably more effective. Surface temperatures recorded for the bare, fabric‐covered, and AOC sheet‐covered car models were 66.0, 59.3, and 51.0 °C, respectively, while the corresponding interior temperatures were 65.6, 59.7, and 48.9 °C. The average solar flux during this interval was 824.4 Wm⁻^2^, with the ambient temperature at 48.6 °C. Unlike the bare and fabric‐covered models, whose temperatures varied in line with solar radiation intensity, the temperature under the AOC sheet‐covered model remained relatively stable, unaffected by changes in solar flux. This stability highlights the AOC sheet's superior performance, particularly under conditions of intense solar radiation, both on the surface and inside the car models.

Although the AOC sheet‐covered car model did not achieve sub‐ambient temperatures, and the temperature differentials between the models were not as pronounced as those depicted in the IR images, the recorded temperature trends demonstrate the AOC sheet's significant cooling effect. This confirms the AOC sheet's potential for widespread application in industries requiring cooling solutions, such as the automotive sector. The larger temperature difference observed between the AOC sheet‐covered car model and the others during high solar flux periods further underscores the sheet's enhanced benefits in extremely hot climates, making it an increasingly attractive option for mitigating heat.

Apart from cooling perspectives, mechanical properties are as important for radiative cooling sheets to be practically applicable. Hence, the AOC sheet's performance under load‐bearing conditions was assessed, as shown in Figure S12 (Supporting Information). The AOC sheet effectively withstands ≈177.8 g of weight without tearing or breaking. This demonstrates its ability to resist deformation under bending or folding, which is relevant to its practical applications, such as installation on curved or irregular surfaces. Further, it is important for a radiative cooling sheet to maintain its cooling performance even when covering outdoor objects for extended periods. To achieve this, temperature resistance and long‐term environmental stability are essential. Therefore, tests were conducted to verify these aspects. An extreme temperature endurance test was conducted to verify the temperature resistance of the AOC sheet. It was heat‐treated by being exposed to a vacuum oven set at 100 °C for 1 week, and changes in the appearance and optical properties of the sheet before and after the test were compared. As shown in Figure S13a (Supporting Information), even after prolonged exposure to extreme temperatures, the AOC sheet exhibited no significant degradation or changes in visual appearance or optical properties.

In order to confirm the environmental stability of the AOC sheet, an accelerated weathering test was conducted. This test involved continuous UV exposure in a 120‐min cycle, where the sample was left still for the first 102 min and then artificial water droplets were sprayed for the afterward 18 min. The test was carried out for a duration corresponding to approximately 6 months in real‐time. After the test, we observed the changes in the appearance and optical properties of the samples before and after the test. The results are indicated in Figure S13b (Supporting Information).

Consequently, the AOC sheet developed in this work possesses outstanding optical properties and cooling capabilities, along with various practical applications, and other performance characteristics that support its use. It shows negligible changes in appearance and optical properties crucial for cooling performance, even after prolonged exposure to extremely high temperatures, intense UV radiation, and weather conditions. The average optical properties of the AOC sheet before and after the tests are shown in Table S4 (Supporting Information). This ensures stable cooling of objects even in harsh environments. Hence, the AOC sheet can maintain effective passive cooling over a long period without degradation, significantly reducing the energy required for cooling. Considering this environmentally friendly perspective, it is expected to be scientifically significant research in cooling industries.

## 3 Conclusion

This study introduces a high‐performance radiative cooling sheet engineered with Al₂O₃‐incorporated organic composite microfibers. The exceptional cooling capability of this sheet is attributed to the optimal optical characteristics of its constituent materials and the structural optimization for radiative cooling. The fabrication process involved electrospinning a solution of PMMA, TPU, and Al_2_O_3_ to create an AOC fabric, followed by hot‐pressing this fabric to produce the final cooling sheet. This sheet demonstrated remarkable sunlight reflectivity at 97.9% and spectral emissivity of 95.2% across the ATW, achieving a simulated cooling power of 120.1 Wm^−2^. These properties enabled the sheet to facilitate 4.9 °C of sub‐ambient cooling during the daytime, surpassing the performance of other covering materials. The AOC sheet's cooling efficiency remained superior irrespective of solar flux, distinguishing it from conventional coverings. In practical applications, such as when applied to a car model, the sheet significantly reduced surface and interior temperatures by 13.1 and 12.7 °C, respectively, in relation to those of an uncovered car. This underlines the sheet's potential across various sectors, especially in automotive industries, where there is a need for heat‐blocking cooling covers. The energy‐free cooling mechanism of the AOC sheet marks a promising advancement for cooling applications, particularly for devices and facilities that traditionally consume significant energy. By implementing this innovative technology, it is possible to significantly decrease energy usage and associated costs, offering a sustainable solution to combat global warming. This breakthrough not only highlights the technical feasibility of passive daytime radiative cooling but also opens avenues for its widespread application, marking a significant stride toward mitigating the impact of global warming.

## 4 Experimental Section

### Materials

PMMA with M_w_ of ≈120 000 was purchased from Sigma–Aldrich, and TPU with M_w_ of ≈130 000 was purchased from BASF Korea. Al_2_O_3_ particles were purchased from Ditto Technology and DMF from Samchun Chemicals, South Korea. All the reagents were used without further purification.

### Experiment

First, PMMA and TPU pellets were dissolved in DMF through magnetic stirring. Al_2_O_3_ particles were added and dispersed in the solution by further stirring when the polymers were fully dissolved. After the experimental optimization of the materials content, PMMA accounted for 20 wt% of the total solution, TPU for 5 wt%, Al_2_O_3_ for 10 wt%, and the remaining 65 wt% was the solvent DMF. The prepared solution was drawn into a syringe with a designed metal nozzle and then electrospun on a 30 cm x 30 cm SUS plate. The electrospinning voltage was fixed to 12.5 kV, and the spinning rate was adjusted to 1 mL h^−1^. The metal nozzle was positioned 15 cm above the SUS plate, and the entire electrospinning was conducted at room temperature and humidity. The AOC fabric produced by electrospinning was detached from the SUS plate and then placed on the stage of the hot‐pressing apparatus, with Teflon paper and rubber sequentially placed on top. Subsequently, the fabric was pressed at 80 °C with a pressure of 2 bars for 20 min, producing the AOC sheet. After the hot‐pressing was completed, the pressure was released, the stage was cooled down to 25 °C and the fabricated AOC sheet was collected.

### Characterization

The sheet fabrication was processed using electrospinning equipment (ESR200R2, NanoNC) and hot‐pressing apparatus (IMDE04, Jung‐and Automatic Technology). The optical properties were characterized using a UV–vis–NIR spectrophotometer (Solidspec‐3700, Shimadzu) and Fourier transform infrared (FTIR) spectroscopy (Nicolet iS50 FTIR spectrometer, Thermo Fisher Scientific). The cooling power was calculated using MATLAB, which uses the principle of radiative cooling. The surface morphology of the AOC sheet was observed using a field emission‐scanning electron microscope (FE‐SEM) (Regulus8100, Hitachi). The temperature of all the samples was measured using a K‐type thermocouple (ST‐50, RKC Instrument), and the corresponding temperature data was collected and recorded on a data logger (OM‐CP‐OCTTEMP‐A, OMEGA). The meteorological data was monitored and logged through a weather station (HD52.3D, Ultrasonic anemometer).

### Statistical Analysis

All experimental measurements were conducted at least three times to ensure reproducibility, and results are presented as mean ± standard deviation (SD). To assess statistical significance, a one‐way analysis of variance (ANOVA) was performed, followed by Tukey's post‐hoc test for multiple comparisons. A significance level of *p* < 0.05 was considered statistically significant. For optical property measurements, the mean values were derived from repeated UV–vis–NIR and FTIR spectroscopic analyses, ensuring consistency across different samples. MATLAB was used for data processing, cooling power calculations, and statistical analysis.

## Conflict of Interest

The authors declare no conflict of interest.

## Supporting information



Supporting Information

## Data Availability

The data that support the findings of this study are available from the corresponding author upon reasonable request.
