# Supplementary material for: Daytime Radiative Cooling Sheet Functionalized by Al2O3‐Assisted Organic Composite
Source: Adv Sci (Weinh). 2025 Feb 3;12(12):2417584. doi: 10.1002/advs.202417584 (PMC11947997; doi:10.1002/advs.202417584)
Supplement: Supplementary file 1 — Supporting Information [file ADVS-12-2417584-s001.pdf]

ADVANCED SCIENCE
Open Access

Supporting Information

for *Adv. Sci.*, DOI 10.1002/advs.202417584

Daytime Radiative Cooling Sheet Functionalized by Al_2O_3-Assisted Organic Composite

*Jaein Park, Dongwoo Chae, Hangyu Lim, Jisung Ha, Seongwoo Park, Hansang Sung, Chanwoong Park and Heon Lee**

Supporting Information

Daytime Radiative Cooling Sheet Functionalized by Al_2O_3-assisted Organic Composite

Jaein Park[a,b], Dongwoo Chae[a], Hangyu Lim[a], Jisung Ha[a], Seongwoo Park[a,b], Hansang Sung[a], Chanwoong Park[a], Heon Lee[*a,b]*

J. Park, D. Chae, H. Lim, J. Ha, S. Park, H. Sung, C. Park, H. Lee
a. Department of Materials Science and Engineering, Korea University, Anam-ro 145, Seongbuk-gu, Seoul, 02841, Republic of Korea
b. ZERC, 620, New Engineering Building, 73-15, Anam-ro, Seongbuk-Gu, Seoul, 02855, Republic of Korea
E-mail: heonlee@korea.ac.kr

Supporting Information 1. Evaluation of Radiative Cooling Performance

The principle of radiative cooling is based mainly on the energy conservation law. The net cooling power as a function of temperature, $P_{Net\ cooling}(T)$, is as follows:

$$P_{Net\ cooling}(T) = P_{rad}(T) - P_{atm}(T_{amb}) - P_{sun} - P_{cond+conv} \qquad (1)$$

P_{rad} refers to the power radiated by the radiative cooler, and it can be expressed as:

$$P_{rad}(T) = \mathrm{A}\int \mathrm{d}\Omega \cos\theta \int_0^\infty \mathrm{d}\lambda I_{BB}(T,\lambda)\varepsilon(\lambda,\theta) \qquad (2)$$

$\int \mathrm{d}\Omega = 2\pi \int_0^{\frac{\pi}{2}} \sin\theta \, d\theta$ in Eq. (2) is the angular integral over a hemisphere, and $I_{BB} = \left(\frac{2hc^2}{\lambda^5}\right)\left(e^{hc/\lambda k_B T} - 1\right)^{-1}$ is the blackbody-specific intensity corresponding to temperature, T, where h represents the Planck's constant, λ is the wavelength, c is the speed of light in vacuum, and k_B indicates the Boltzmann constant. $\varepsilon(\lambda,\theta)$ is the spectral and angular emissivity of the cooler, and A is the surface area.

P_{atm} represents the atmospheric radiation power, and is expressed with respect to the ambient temperature as follows:

$$P_{atm}(T_{amb}) = \mathrm{A}\int \mathrm{d}\Omega \cos\theta \int_0^\infty \mathrm{d}\lambda I_{BB}(T,\lambda)\varepsilon(\lambda,\theta)\varepsilon_{atm}(\lambda,\theta) \qquad (3)$$

According to Kirchhoff's law of thermal radiation, the absorptivity of a material at a given wavelength is equal to its emissivity at the same wavelength, under thermodynamically equilibrium conditions. $\varepsilon_{atm}(\lambda, \theta) = 1 - t(\lambda)^{1/\cos\theta}$ in Eq. (3) represents the atmospheric emissivity, wherein $t(\lambda)$ is the atmospheric transmittance in the zenith direction.

P_{sun} refers to the incident power absorbed from the sun, and it is expressed as follows:

$$P_{sun} = \mathrm{A}\int_0^\infty \mathrm{d}\lambda \varepsilon(\lambda, \theta_{sun}) I_{AM1.5}(\lambda) \quad (4)$$

Here, $I_{AM1.5}$ is the intensity of solar illumination.

$P_{cond+conv}$ is the non-radiation power, which corresponds to the sum of conduction and convection powers. It can be expressed based on Newton's law as follows:

$$P_{cond+conv}(T, T_{amb}) = Ah_c(T_{amb} - T) \quad (5)$$

Here, h_c is the non-radiative heat transfer coefficient.

In total, $P_{Net\ cooling} > 0$ is the essential condition to be met to perform a positive degree of radiative cooling, and the temperature where $P_{Net\ cooling} = 0$ represents the steady-state temperature of the material, T_s.

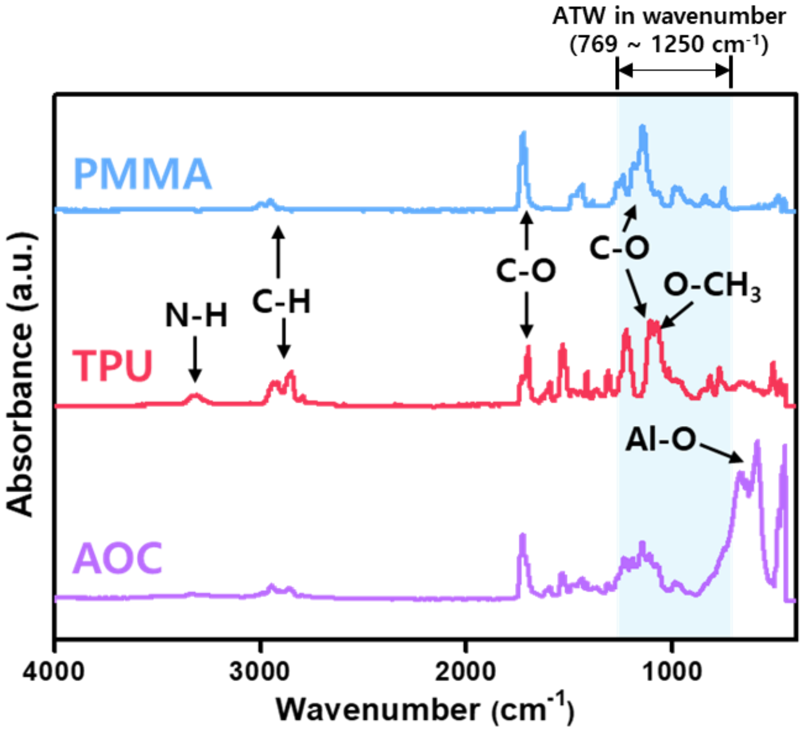

Figure S1. ATR-FTIR analysis for PMMA, TPU, and AOC sheet. Spectral data were obtained from three independent measurements (n = 3) and are presented as mean ± SD.

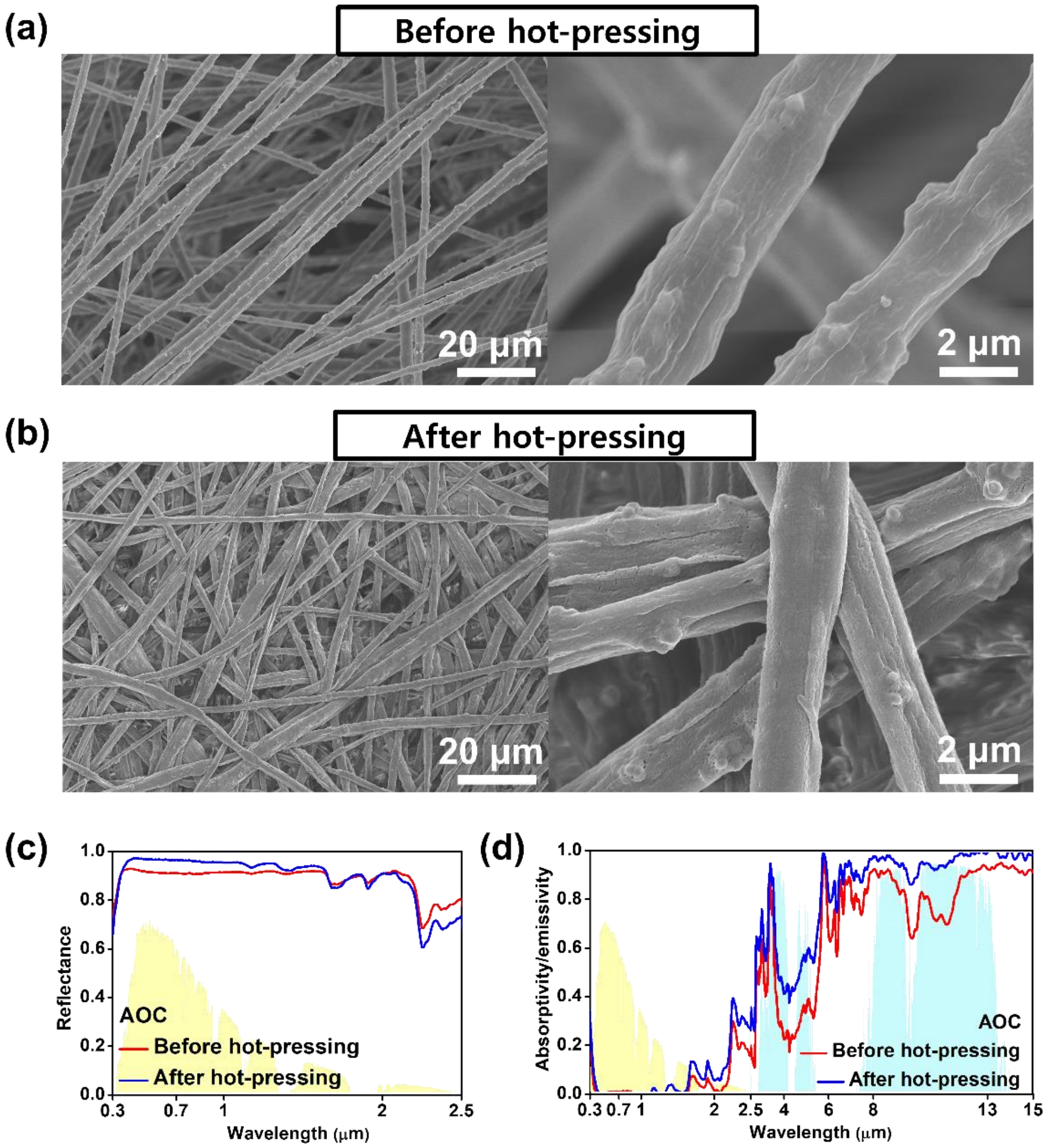

Figure S2. Low and high-resolution SEM images of the AOC a) before hot-pressing and b) after hot-pressing, measured c) solar reflectance and d) spectral absorptivity/emissivity of the AOC sheet before and after hot-pressing. Statistical significance was determined using an independent t-test ($p < 0.05$).

Table S1. Averaged optical properties of the AOC sheet before and after hot-pressing.

Hot-pressing	Thickness (μm)	Solar reflectance(%)	Emissivity in ATW (%)
Before	390	91.1	82.3
After	260	95.1	94.7

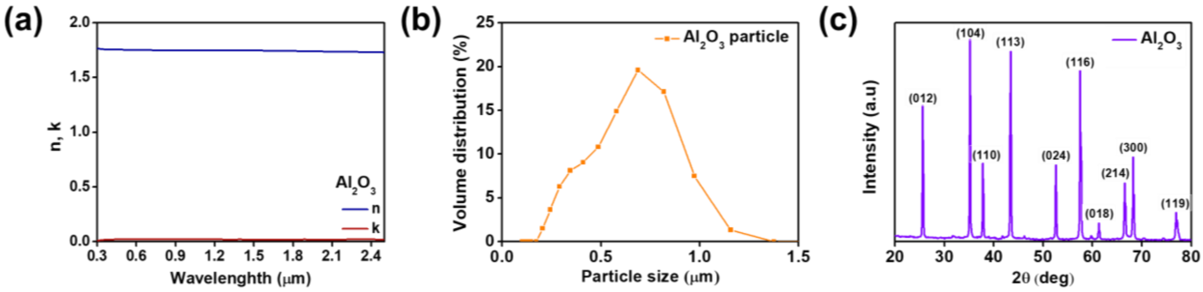

Figure S3. a) Refractive index and extinction coefficient of Al_2O_3 particles throughout solar wavelengths (0.3–2.5 μm), b) particle size distribution derived by volume percentage, and c) XRD 2θ plots of the used Al_2O_3 particles.

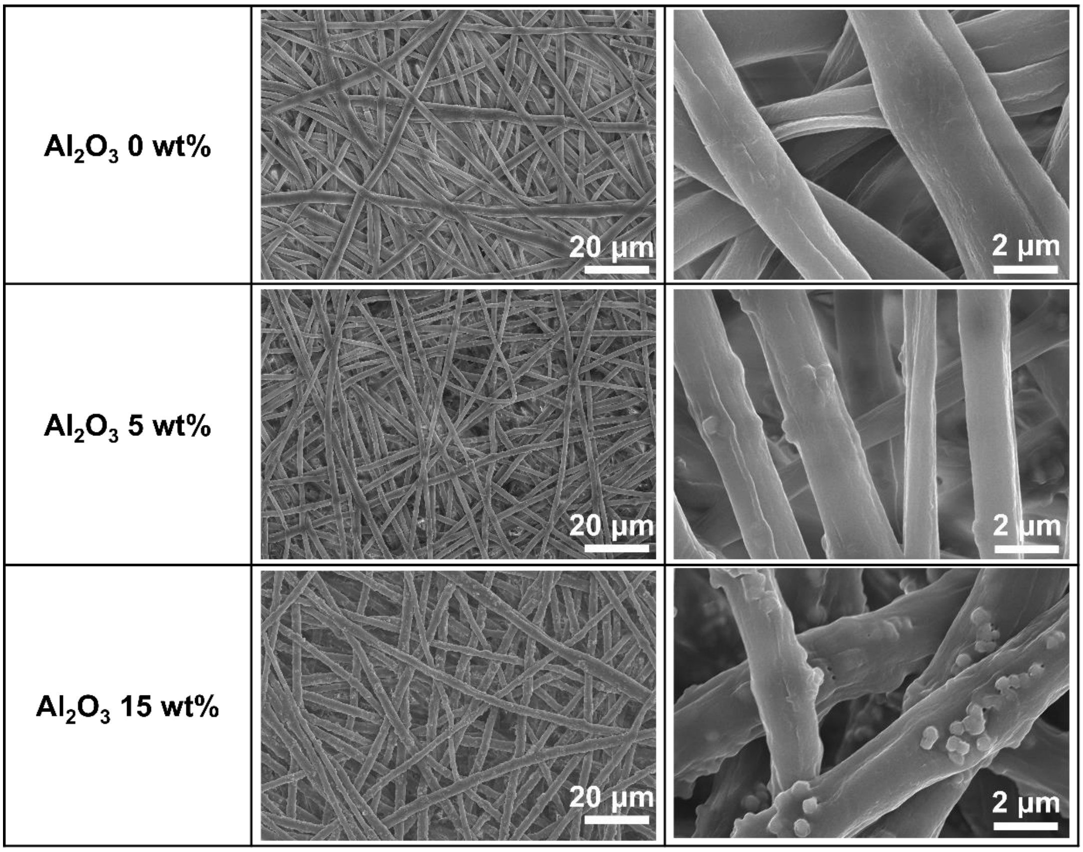

Figure S4. Low and high-resolution SEM images of the fabricated AOC sheet incorporating 0, 5, and 15 wt% of Al_2O_3.

From the images, it was observed that no shape of particles was shown in the sheet without the Al_2O_3, while the number of particles embedded on the fibers in the microstructure increased with the increment in Al_2O_3 content, considering **Figure 2b-c**.

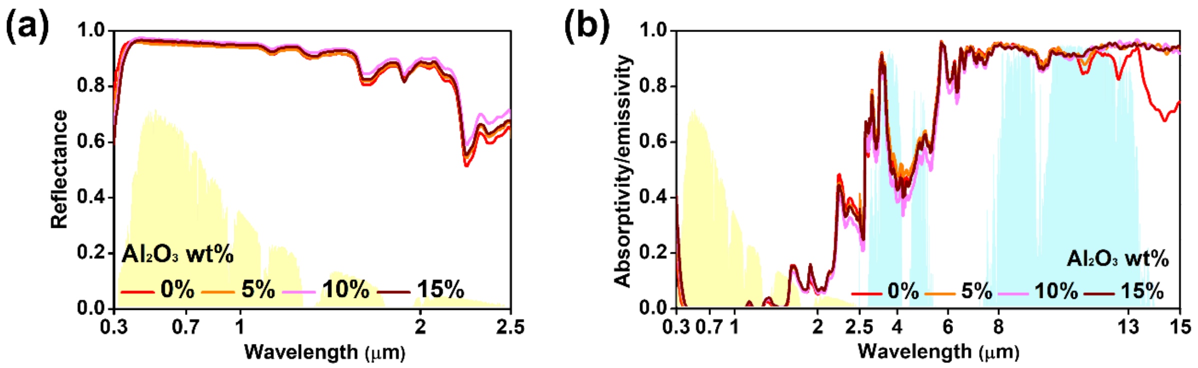

Figure S5. a) Measured solar reflectance and b) spectral absorptivity/emissivity of the AOC sheet with 0, 5, 10, and 15 wt% of Al_2O_3. Optical properties were measured in three independent experiments (n = 3) per sample and are presented as mean ± SD. One-way ANOVA with Tukey's post-hoc test ($p < 0.05$) was conducted to evaluate the effect of varying Al_2O_3 content.

Table S2. Averaged optical properties of the AOC sheet with 0, 5, 10, and 15% of Al_2O_3.

Al_2O_3 wt%	Thickness (µm)	Solar reflectance(%)	Emissivity in ATW (%)
0	260	93.7	94.0
5	260	93.3	94.2
10	260	95.0	95.8
15	260	94.2	93.6

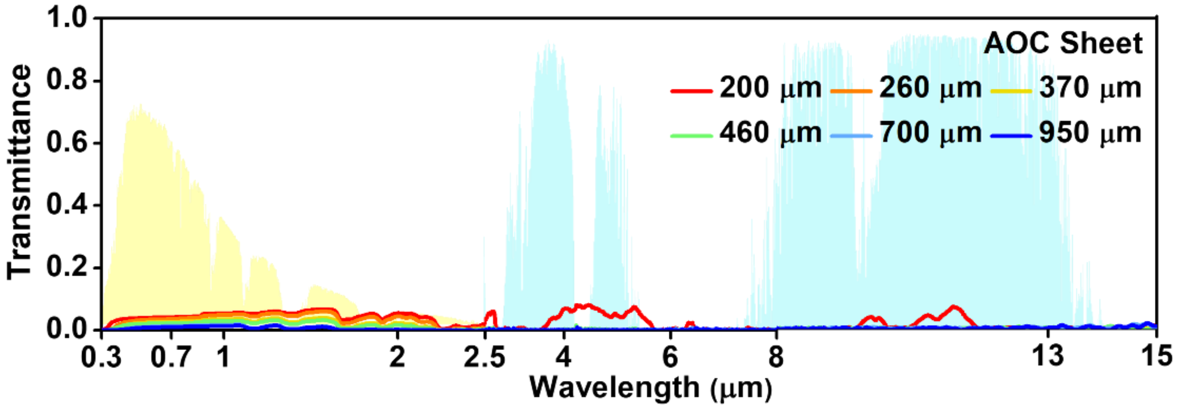

Figure S6. Spectral transmittance of the AOC sheet with different thicknesses.

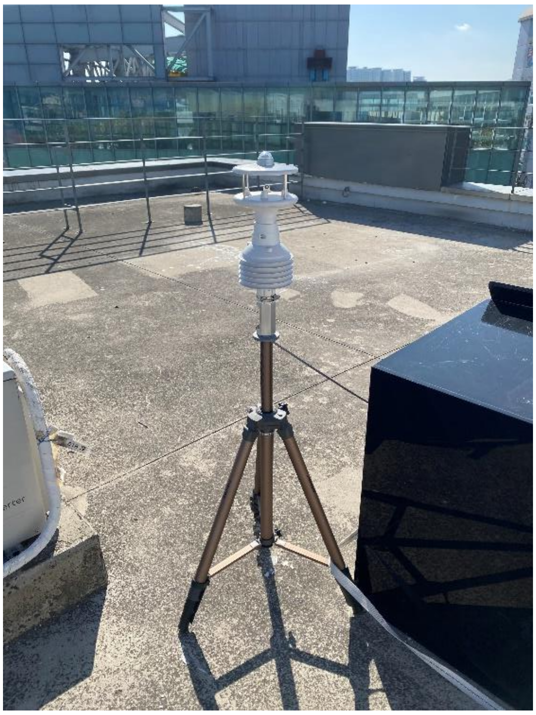

Figure S7. Photo image of the weather station used for collecting the weather data.

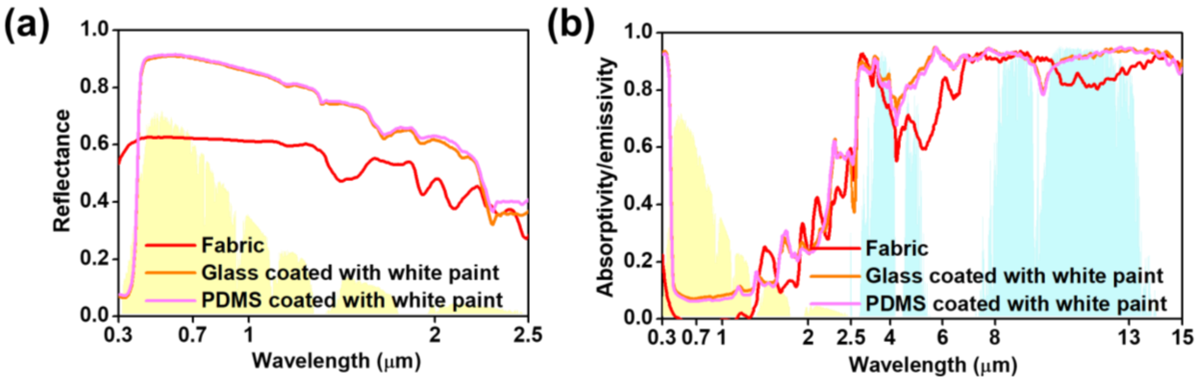

Figure S8. a) Measured solar reflectance and b) spectral absorptivity/emissivity of the comparative samples: Bare fabric, CW-paint-coated glass and PDMS. Reflectance and emissivity data were obtained from three independent trials ($n = 3$) and reported as mean ± SD. Statistical significance among different material groups was assessed using one-way ANOVA followed by Tukey's post-hoc test ($p < 0.05$).

Table S3. Averaged optical properties of the fabric, CW-paint-coated glass and PDMS used during the outdoor temperature measuring experiment.

Sample		Solar reflectance (%)	Emissivity in ATW (%)
Fabric		60.2	87.1
CW painted on	Glass	82.3	90.9
	PDMS	82.9	90.7

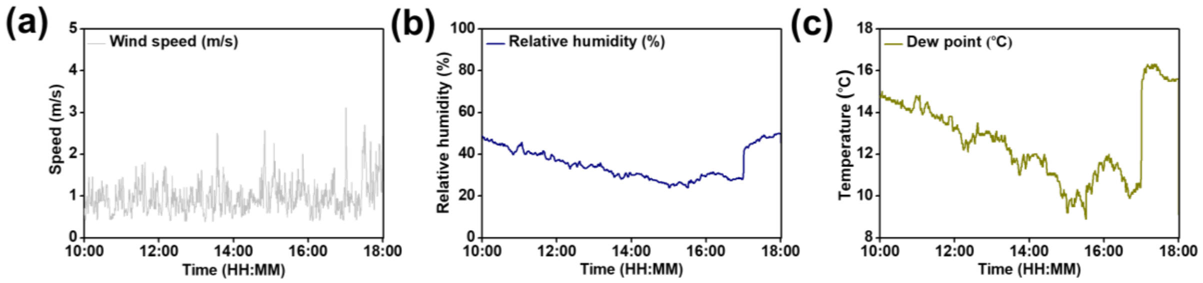

Figure S9. Environmental conditions during the outdoor temperature measuring experiment: a) wind speed, b) relative humidity, and c) dew point corresponding to **Figure 4.**

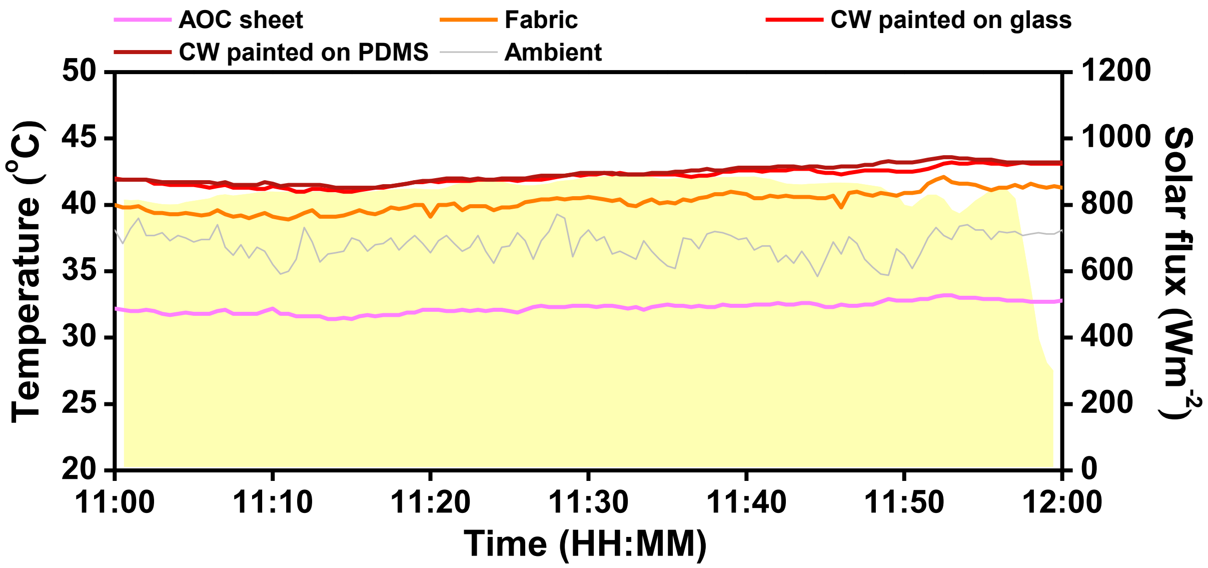

Figure S10. Temperature variations during the period of highest solar flux.

The temperature measurement results from 11:00 to 12:00 presents the shortened period of the outdoor experiment corresponding to **Figure 4.** During this time, with 831.7 Wm^{-2}, the average temperature of the AOC sheet was 32.3 °C, while the temperatures of the fabric, CW-paint-coated glass and PDMS were 40.2, 42.1, and 42.3 °C, respectively. The average ambient temperature was 37 °C. This indicates that the AOC sheet can perform passive cooling both under extreme sunlight and during periods with less or negligible sunlight. Temperature data were obtained from three independent trials (n = 4) and are presented as mean ± SD. Statistical significance among different samples was analyzed through one-way ANOVA with Tukey's post-hoc test ($p < 0.05$).

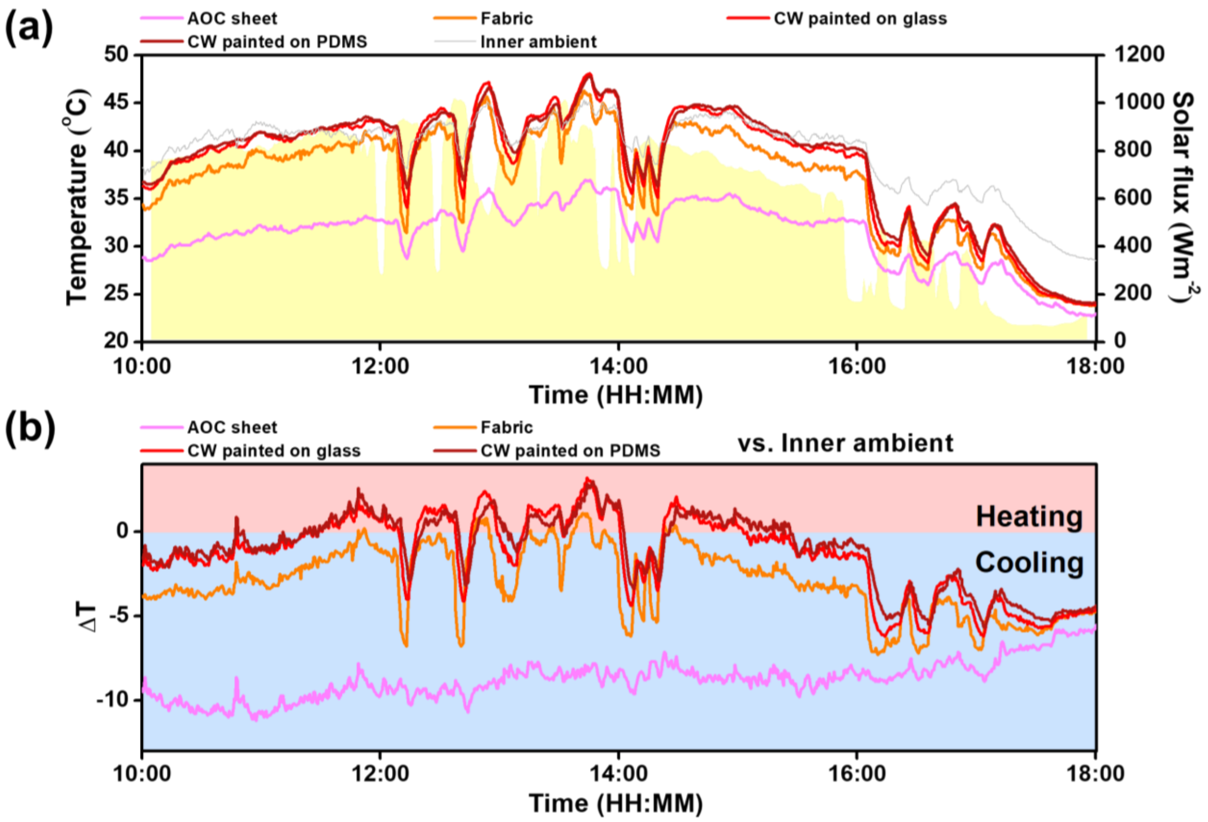

Figure S11. a) Measured temperature of the AOC sheet and the comparative samples with inner ambient temperature and b) temperature difference between the measuring samples and the inner ambient.

Because the inner ambient temperature was measured in an enclosed environment free from wind, convection and conduction in relation to the outer ambient, it was recorded at a higher temperature. Consequently, the temperature difference among the samples under the same conditions became more pronounced. The average inner ambient temperature during the outdoor temperature measuring experiment was 40.0 °C. Accordingly, the AOC sheet, fabric, CW paint-coated glass and PDMS showed temperatures lower by 8.8, 3.0, 1.4, and 1.1 °C, respectively, in relation to the inner ambient. From 11:00 to 12:00, corresponding to the period when solar flux was intense, the logged inner ambient temperature was 42.0 °C, and thereby the AOC sheet, fabric, CW-paint-coated glass and PDMS exhibited 10.8, 3.0, 1.0, and 1.1 °C of sub-ambient cooling. Since the AOC sheet exhibited at least 7.8 °C lower temperature relative to the comparative group along with a far more prominent sub-ambient

cooling during the intense solar flux. It was confirmed that the AOC sheet consistently provided adequate cooling regardless of the solar intensity. Three independent trials (n = 4) were conducted, with data reported as mean ± SD. One-way ANOVA with Tukey's post-hoc test ($p < 0.05$) was used to assess statistical differences.

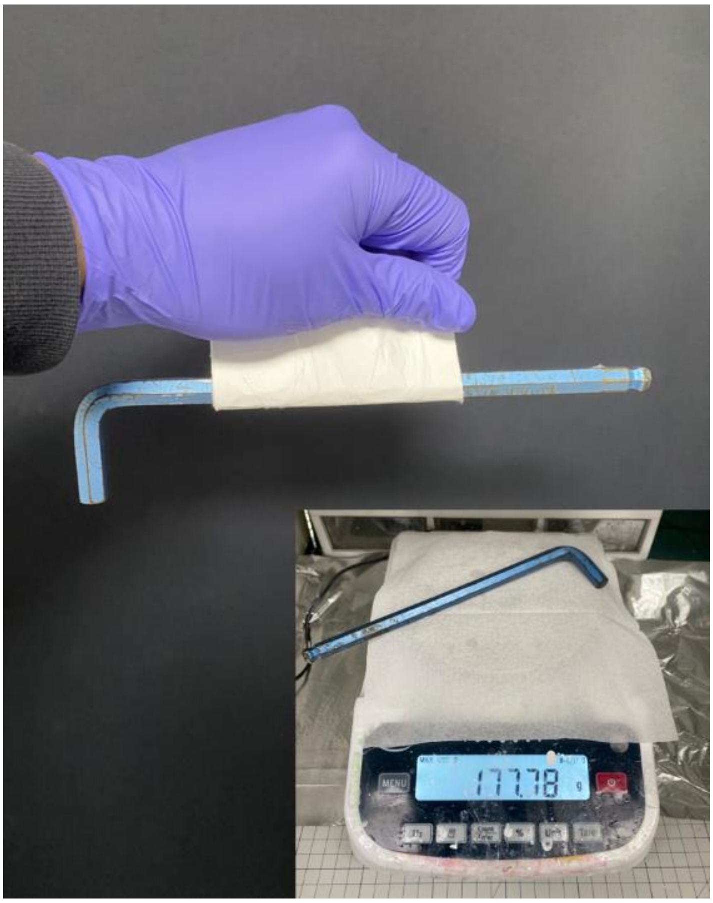

Figure S12. Photo image of the load-bearing experiment on the AOC sheet, showing the sheet supporting a metal rod with an actual weight of 177.8 g as indicated in the inset.

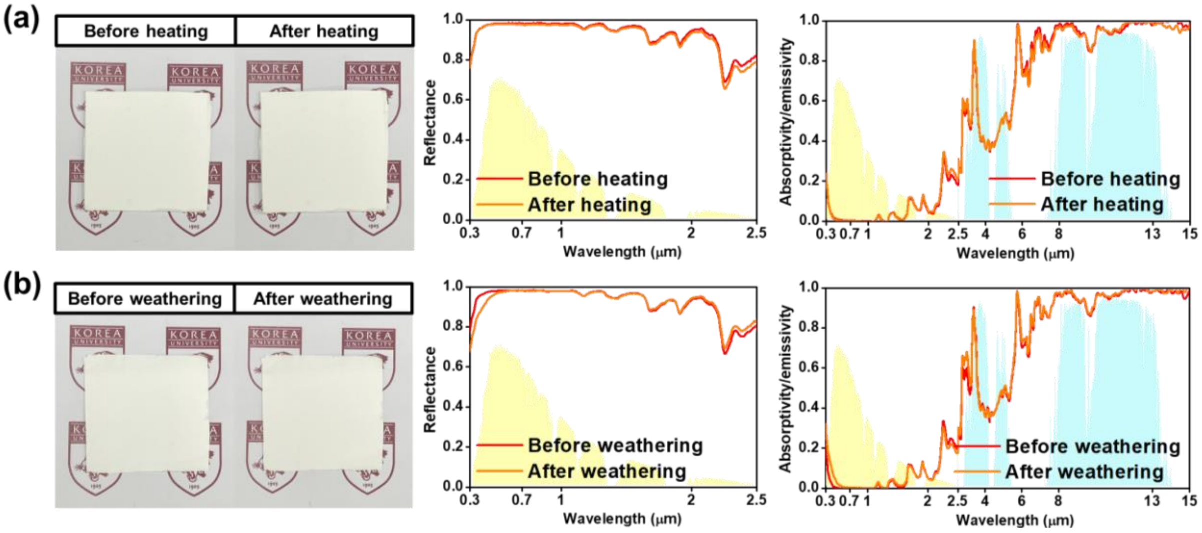

Figure S13. Photo images and measured optical properties of the AOC sheet before and after a) heat treatment and b) weathering test. Solar reflectance and emissivity data before and after tests were obtained from three independent measurements (n = 3) per each sample, and are presented as mean ± SD. Statistical significance was determined using an independent t-test ($p < 0.05$).

Table S4. Average optical properties of the AOC sheet before and after heat treatment and weathering test.

Treatment		Solar reflectance (%)	Emissivity in ATW (%)
Heating	Before	97.0	95.6
	After	96.5	95.6
Weathering	Before	96.9	95.9
	After	96.1	96.5
